# *Cleistocalyx nervosum* var. *paniala* berry extract and cyanidin‐3‐glucoside inhibit hepatotoxicity and apoptosis

**DOI:** 10.1002/fsn3.3975

**Published:** 2024-01-19

**Authors:** Pasitta Panritdum, Chawanphat Muangnoi, Siriporn Tuntipopipat, Somsri Charoenkiatkul, Monruedee Sukprasansap

**Affiliations:** ^1^ Graduate student in Master of Science Program in Nutrition, Faculty of Medicine Ramathibodi Hospital and Institute of Nutrition Mahidol University Bangkok Thailand; ^2^ Cell and Animal Model Unit, Institute of Nutrition Mahidol University Nakhon Pathom Thailand; ^3^ Institute of Nutrition Mahidol University Nakhon Pathom Thailand; ^4^ Food Toxicology Unit, Institute of Nutrition Mahidol University Nakhon Pathom Thailand

**Keywords:** chronic liver diseases, *Cleistocalyx nervosum* var. *paniala* fruit, endogenous antioxidant enzymes, hepatotoxicity, HepG2 cells, oxidative toxicity

## Abstract

Excessive oxidative toxicity in liver cells is a significant risk factor that can cause cellular injury, leading to the development of chronic liver disease (CLD). Natural anthocyanins have been shown to prevent the harmful effects of oxidative toxicity in mammalian cells. Ripe *Cleistocalyx nervosum* var. *paniala* berry fruits are rich in anthocyanins, which have been reported to possess many health benefits. Therefore, this study examined the protective effect of ethanolic fruit extract of *C. nervosum* var. *paniala* (CNPE) against hydrogen peroxide (H_2_O_2_)‐induced oxidative damage and cell death in human hepatoma HepG2 cells. Results showed that CNPE had strong antioxidant capabilities and high amounts of total phenolics and anthocyanins. HPLC analysis showed that CNPE consists of cyanidin‐3‐glucoside (C3G). Our investigations found that HepG2 cells pretreated with CNPE or anthocyanin C3G inhibited H_2_O_2_‐induced cellular damage and apoptosis by increasing the viability of cells, the expression of antiapoptotic Bcl‐2 protein, and the activities of cellular antioxidant enzymes, namely SOD, CAT, and GPx. Moreover, both CNPE and C3G significantly suppressed expression of apoptotic proteins (Bax and cytochrome c) and the activities of cleaved caspase‐9 and caspase‐3 caused by H_2_O_2_. Our results indicate that CNPE and C3G can suppress H_2_O_2_‐induced hepatotoxicity and cell death through stimulation of endogenous antioxidant enzyme activities and inhibition of apoptosis pathway in HepG2 cells. These findings might support development of CNPE as an alternative natural product for preventing CLD.

## Graphical abstract

Schematic view of the proposed effect and mechanism of *Cleistocalyx nervosum* var. *paniala* ethanolic extract (CNPE) protect against H_2_O_2_‐induced oxidative toxicity and apoptosis in HepG2 cells via inhibition of the apoptosis pathway and activation of cellular antioxidant enzymes activities.

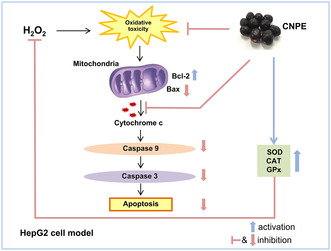

## 1 INTRODUCTION

Chronic liver diseases are a major cause of mortality worldwide, specifically fatty liver disease, hepatitis leading to cirrhosis and liver cancer, etc. (Embade & Millet, 2017). In many cases, liver cells can repair the damage that occurs in the early stages, but long‐term damage in liver cells can trigger fibrosis. This event can lead to chronic inflammation and cirrhosis in liver cells which increases the risk of liver cancer. Hepatocellular carcinoma is the common cause of approximately 90% of cases of primary liver cancer (Balogh et al., 2016). It associates with chronic liver damage, leading to liver fibrosis and cirrhosis, which contribute to liver cancer via mitochondrial damage and oxidative stress caused by increased reactive oxygen species production (Yuan et al., 2018). Generally, mitochondria play an important role in cellular bioenergetics. Oxidative toxicity is produced during mitochondrial oxidative metabolism as a by‐product of mitochondrial respiration. The radical species also play an important role in cell proliferation and differentiation including superoxide anions and hydroxyl radicals (Ray et al., 2012; Yao et al., 2016). In liver cells, their functions are responsible for metabolizing endogenous and exogenous compounds. Therefore, the liver is a targeted organ for the toxic action of xenobiotics or their reactive metabolites. During metabolism in hepatocytes, xenobiotics can generate free radicals (Chiu et al., 2013). The liver has high metabolic activity and receives toxicity from a variety of drugs and environmental contaminants. They can also be another cause of oxidative‐induced toxicity mechanisms related to continuous liver injury, affecting hepatocytes and activating liver macrophages (Kupffer cells [KCs]) to produce more accumulated collagen, leading to fibrosis and, eventually, cirrhosis (Ma et al., 2016). Furthermore, an excess of radicals contributes to the accumulation of oxidative stress status, leading to DNA, protein, and lipid oxidation in liver cells (Martindale & Holbrook, 2002). Prolonged oxidative stress associates with mitochondria and cellular damage or dysfunction, contributing to the apoptotic signal and cell death pathways. Mechanisms of apoptosis are manifold and diverse, with numerous apoptotic molecule markers related to the mitochondrial apoptotic pathway, such as caspase‐9, caspase‐3, cytochrome c (cyt‐c), the pro‐apoptotic protein Bax, and the anti‐apoptotic protein Bcl‐2 families which subsequently lead to apoptotic cell death (Jiang et al., 2014; Jiao et al., 2017). Therefore, the reduction of oxidative toxicity‐induced cell injury and death is a critical approach for preventing various non‐communicable diseases (NCDs), such as the chronic liver diseases. These events are involved in the activities of cellular antioxidant defense systems such as superoxide dismutase (SOD), catalase (CAT), glutathione peroxidase (GPx), etc., which regulate the balance of free radicals and oxidative stress within liver cells to safeguard against the harmful effects of prolonged oxidative toxicity (Albrahim & Alonazi, 2022; Ezhilarasan, 2018; Kalpana et al., 2021).

Natural antioxidants, especially anthocyanins, can effectively protect against the deleterious effects of oxidative toxicity in cells (Fallah et al., 2020). Anthocyanins are naturally occurring purple‐blue pigments belonging to the group of flavonoids, which includes several types of anthocyanidins including cyanidin, delphinidin, pelargonidin, peonidin, petunidin, and malvidin (Martín et al., 2017). They are found in plants, particularly berry fruits (Mattioli et al., 2020). Anthocyanins have been reported to benefit health, particularly in the prevention of various diseases associated with oxidative toxicity or inflammation such as dyslipidemia, cardiovascular, and neurodegenerative diseases (Xu et al., 2020). The antioxidant properties of anthocyanins have been demonstrated by in vitro and in vivo experiments, such as where anthocyanins from *Hibiscus syriacus* L. decreased hydrogen peroxide (H_2_O_2_)‐induced excessive oxidative toxicity and prevented apoptosis in HaCaT keratinocytes by stimulating antioxidant pathway (Molagoda et al., 2020). In addition, anthocyanins have played a role in the prevention of cancer in in vivo study, for example, the protective effects of black currant extract rich in anthocyanins on diethylnitrosamine in rats, where it reduced the risk of abnormal proliferation before cancer formation via the upregulation of Bcl‐2‐associated X protein (Bax) and downregulation of B‐cell lymphoma‐2 (Bcl‐2) expression (Bishayee et al., 2011).

*Cleistocalyx nervosum* var. *paniala*, natively known as Ma‐kiang, is found in Southeast Asia, especially in the Northern region of Thailand. It belongs to the family Myrtaceae. A ripe fruit of *C. nervosum* var. *paniala* is red‐purple to dark‐purple color which indicates the abundance of anthocyanin pigments (Poontawee et al., 2016). Previous studies found that cyanidin‐3‐glucoside (C3G) is the major anthocyanin in the ripe fruit of *C. nervosum* var. *paniala* (Jansom et al., 2008; Sukprasansap et al., 2017). It offers enhanced antioxidant, anti‐inflammatory (Tan et al., 2019), anticancer (Cho et al., 2017), and neuroprotective properties (Sukprasansap et al., 2020). Furthermore, previous studies have demonstrated that C3G protected HepG2 cells from oxidative damage by H_2_O_2_ via the direct decrease of reactive radicals. This compound could upregulate expression of antioxidant enzymes, such as SOD, CAT, and GPx; meanwhile, it downregulated the protein expression levels of pro‐apoptotic Bax and upregulated the protein expression levels of anti‐apoptotic Bcl‐2 in HepG2 cells (Sukprasansap et al., 2020; Tan et al., 2020). Based on these scientific data, it is possible to utilize the ripe fruit of *C. nervosum* var. *paniala* ethanolic extract (CNPE) to inhibit oxidative damage in human liver cells. This research also aimed to investigate the protective effect of CNPE against oxidative toxicity and apoptotic cell death caused by H_2_O_2_ in human hepatoma HepG2 cells.

## 2 MATERIALS AND METHODS

### 2.1 Reagents

Dulbecco's modified Eagle's medium (DMEM) (Life Technologies Corporation, Grand Island, NY 14072, USA). The primary antibodies Bax (#2772), Bcl‐2 (124) (#15071), Cytochrome c (D18C7) (#11940) were purchased from Cell Signaling Technology (Danvers, MA, USA). The secondary antibodies anti‐rabbit IgG, HRP‐linked Antibody (#7074) anti‐mouse IgG, HRP‐linked Antibody (#7076), and β‐actin (13E5) (#4967) were purchased from Cell Signaling Technology (Danvers, MA, USA). Ethanol and methanol were purchased from RCI Labscan Limited (Bangkok 10330, Thailand). Folin & Ciocalteu's phenol reagent, 2,4,6‐Tris (2‐pyridyl)‐s‐triazine (TPTZ), 2,2‐diphenyl‐1‐picrylhydrazyl (DPPH) and (±)‐6‐Hydroxy‐2,5,7,8‐tetramethylchromane‐2‐carboxylic acid (Tro‐lox), cyanidin‐3‐glucoside (C3G; purity ≥99%) were purchased from Sigma‐Aldrich (St. Louis, MO, USA).

### 2.2 Preparation and extraction of samples

The ripe *C. nervosum* var. *paniala* fruits were collected from the cultivated area of the Plant Genetic Conservation Project under the Royal Initiation of Her Royal Highness Princess Maha Chakri Sirindhorn (RSPG), Lampang province, Thailand, during July–August 2018. The *C. nervosum* var. *paniala* was identified as the scientific name by Assistant Professor Dr. Thaya Jenjittikul (Department of Plant Science, Faculty of Science, Mahidol University, Thailand). Voucher specimen was No. 9428, which was deposited at Suan Luang Rama IX Herbarium, Bangkok, Thailand. The fruits were washed with tap water and then DI‐water, stem was removed and air dried. The pulp and seed were separated and weighed. Next, the pulp was freeze‐dried and ground to powder. It was then packed in vacuum‐aluminum foil and kept at −20°C until analysis. The powder samples were extracted with 95% ethanol at the ratio 1 g of sample: 15 mL of extract solvents. The sample was mixed by vortex mixer and kept at 25°C, overnight (16–18 h). The sample was then sonicated in an ultrasonic bath (DAIHAN Scientific, Gangwon‐do, Korea) at 25°C for 10 min and mixed by vortex mixer for 1 min. Next, the extract was centrifuged at 4140 *g* for 10 min (Hettich® Instruments, Rotina 38R, Germany). The supernatant was transferred to flat bottom flask. After that, it was evaporated under vacuum at 35–40°C until dry, then solubilized with 2 mL of extract solvent and transferred to amber glass vial in darkness. The extract was blown with nitrogen gas until dry at room temperature, then kept at −20°C until analysis. Percentage of yield was 27.11 ± 2.77%. This extract is hereafter referred to as CNPE.

### 2.3 Determination of antioxidant properties

#### 2.3.1 The 2,2‐diphenyl‐1‐picrylhydrazyl (DPPH) assay

This assay is based on measuring the ability of antioxidants to scavenge DPPH radicals. The procedure was described by Sukprasansap et al. (2017). Briefly, the sample extracts (20 μL) at concentration 0.125–12.5 mg/mL were added to a 96‐well plate and added the DPPH reagent in 90% ethanol (200 μL). Then, the plate was incubated in the dark at room temperature for 30 min. Absorbance was measured at 520 nm using a microplate reader (BioTek® Instruments, Vermont, USA). Trolox (TE), a hydrophilic analog of vitamin E, was used as a standard equivalent. Data were calculated with standard curve of Trolox and expressed as the inhibitory concentration (IC)‐50 value by the linear equation.

#### 2.3.2 Ferric reducing antioxidant power (FRAP) assay

Another method for detecting antioxidant power capacity is based on the reduction of a ferric 2,4,6‐tripyridyl‐s‐triazine complex (Fe^3+^‐TPTZ) to the ferrous form (Fe^2+^‐TPTZ). The sample extracts (20 μL) were added to a 96‐well plate followed by 150 μL of FRAP reagent (TPTZ solution and ferric chloride in acetate buffer). Then, the plate was incubated in the dark at room temperature for 8 min. Absorbance of sample was measured at 600 nm using a microplate reader. Results were calculated with standard curves of TE solution and expressed as μmol TE/g dry weight (DW) (Sukprasansap et al., 2017).

### 2.4 Total phenolic content

Total phenolic content was estimated using the Folin–Ciocalteu colorimetric method. The method was slightly adapted from Nantacharoen et al. (2022). The sample extract (10 μL) was added to a 96‐well plate followed by 160 μL of deionized water. Next, the Folin–Ciocalteu reagent (10 μL) and sodium carbonate solution (20 μL) were added, and then incubated in the dark at room temperature for 30 min. The absorbance was measured at 750 nm using a microplate reader. Results were calculated with standard curves of gallic acid and expressed as mg gallic acid equivalent (GAE)/100 g DW.

### 2.5 Total anthocyanin content

The total anthocyanin content was performed according to the method reported by Sukprasansap et al., 2017 and Srimard et al., 2022. Briefly, the anthocyanins were extracted with acidified methanol (methanol and 1 M HCl, 85:15, v/v) at the ratio 1 g of sample: 10 mL of extract solvent for 10 min. The sample was mixed with vortex mixer. Then, sample extract (100 μL) was added to a 96‐well plate. After that, the plate was incubated in the dark at room temperature for 15 min. The absorbance was measured at 525 nm using a microplate reader. Total anthocyanin content was calculated using a calibration curve of C3G as a reference. Results were expressed as mg C3G equivalent/100 g DW.

### 2.6 Identification of C3G in CNPE

The C3G content in CNPE was determined by high‐performance liquid chromatography (HPLC) technique using a Zorbax Eclipse XDB‐C18 column (4.6 × 150 mm) and diode array detection with SHIMADZU LC‐10 series (Shimadzu Scientific Instruments, MA, USA), according to the method described by Sukprasansap and colleagues. The mobile phases consist of A (2% acetic acid in water) and B (absolute methanol) at a flow rate of 1.0 mL/min. The 20 μL of sample was directly injected into the column, thermostated at 35°C. The C3G was measured at 520 nm using UV‐DAD. The content of C3G was quantified by comparing retention times and spectral absorption with C3G standard compound (purity ≥99%, Sigma‐Aldrich). Quantification of C3G in the CNPE was expressed as mg/100 g DW.

### 2.7 Cell culture

The human hepatoma HepG2 cell line was obtained from the American Type Culture Collection (ATCC; Manassas, VA, USA). HepG2 cells were cultured in a complete medium (DMEM supplemented with 10% (v/v) of fetal bovine serum (FBS), 1% (v/v) of penicillin–streptomycin). The HepG2 cells were grown in 75‐T flask (75cm^2^ cell culture flask) and maintained with a complete medium at 37°C in a humidified incubator atmosphere containing 5% CO_2_ and sub‐cultured every 5–7 days. Cells were grown to 80–90% confluence for the experiments.

### 2.8 Cell viability assay

Cell viability was determined by 3‐(‐4,5‐dimethylthiazol‐2‐yl)‐2, 5 diphenyltetra‐zolium bromide tetrazolium (MTT) assay. The cells were seeded at a density of 5 × 10^4^ cells/well in 96‐well plates for 24 h. Next, to evaluate the cytotoxicity of samples and select the non‐toxic concentrations to use for the subsequent analysis, the cells were washed with serum‐free medium, then added serum‐free medium containing various concentrations of CNPE (10–200 μg/mL) or H_2_O_2_ (100–700 μM) or C3G (5–40 μM) for 24 h. The 0.2% DMSO was used as a vehicle control group. For the protective effect experiment, the cells were pretreated with CNPE (1–100 μg/mL) or C3G at 10 μM (used as a positive control), followed by 300 μM H_2_O_2_ for 24 h. After incubation, cells were washed with PBS, and MTT solution at 5 mg/mL (20 μL) was added to each well and cultured for 4 h at 37°C. Subsequently, the MTT solution was removed and DMSO (100 μL) was added to each well. The optical density was measured at 540 nm using a microplate reader. Results were presented as the percentage of cell viability in comparison with the control group.

### 2.9 Caspase‐9 and caspase‐3 activities assay

Determination of cleaved caspase‐9 and caspase‐3 activities was performed with the caspase activity assay kit from Cayman Chemical (Ann Arbor, MI, USA). HepG2 cells were seeded in 6‐well plates at a 1.2 × 10^6^ cell/well. After incubation for 24 h, the cells were washed with the serum‐free medium and added to serum‐free medium containing various concentrations (1–100 μg/mL) of CNPE (treatment group) or 10 μM of C3G (used as a positive control) for 24 h. Cells were washed once with serum‐free medium. Then, the cells were incubated with 300 μM H_2_O_2_ for 24 h. After incubation, the cells were washed with PBS and added to the lysis buffer (0.5% Triton X‐100 in PBS). The cells were centrifuged at 17,320 *g* at 4°C for 10 min. The supernatants were collected to determine cleaved caspase‐9 and caspase‐3 activities using commercial assay kits. The cleaved caspase activities were determined by fluorescence intensity (excitation at 485 nm and emission at 535 nm) using a CLARIOStar microplate reader (BMGLABTECH, Offenburg, Germany).

### 2.10 Western blot analysis

The HepG2 cells were seeded in 6‐well plates at a 1.2 × 10^6^ cell/well. After incubation for 24 h, the cells were washed with serum‐free medium and added to serum‐free medium containing various concentrations (1–100 μg/mL) of CNPE (treatment group) or 10 μM of C3G (used as a positive control) for 24 h. Cells were washed once with serum‐free medium, then incubated with 300 μM H_2_O_2_ for 24 h. After incubation, the treated cells were washed once with cold PBS and added to cold lysis buffer (RIPA buffer contained phosphatase and protease inhibitor) and shaken at 4°C for 30 min. Cell lysate was collected after centrifugation at 17,320 *g* at 4°C for 10 min. Only the supernatant of cell lysate was collected and transferred into new tubes. Protein concentrations were determined using bicinchoninic acid (BCA) protein assay kit (Thermo Scientific, Rockford, USA), which is used to quantify the total protein in a sample. Equal amounts of protein samples (40 μg) were separated by 10% SDS‐PAGE and the separated proteins were transferred to nitrocellulose membranes (Amersham™ Protran®; Sigma‐Aldrich). After transfer, the protein membrane was blocked with 5% non‐fat dried milk for 1 h at room temperature, Then, the membrane was washed with tris‐buffered saline tween (TBST), followed by incubation with specific primary antibodies (anti‐Bax 1:1000, anti‐Bcl‐2 1:1000, anti‐cytochrome c 1:1000 [Cell Signaling Technology, Danvers, MA, USA]) overnight at 4°C, then incubated with secondary horseradish peroxidase (HRP)‐conjugated anti‐rabbit/mouse 1:2000 (Cell Signaling Technology, Danvers, MA, USA) for 2 h at room temperature. Next, the membranes were incubated with chemiluminescence substrate and then exposed to X‐ray film. Results were expressed as relative ratio of band intensity between targeted proteins and β‐actin.

### 2.11 Antioxidant enzyme activities assay

The activity of CAT, GPx, and SOD in HepG2 cells was determined using commercial assay kits from Cayman Chemical (Ann Arbor, Michigan, USA). HepG2 cells were seeded in 6‐well plates at a 1.2 × 10^6^ cell/well. After incubation for 24 h, the cells were washed with serum‐free medium and added to serum‐free medium containing various concentrations (1–100 μg/mL) of CNPE (treatment group) or 10 μM of C3G (used as a positive control) for 24 h. The cells were washed once with serum‐free medium, and then incubated with 300 μM H_2_O_2_ for 24 h. After incubation, the cells were washed with PBS and added the lysis buffer (0.5% Triton X‐100 in PBS). The cells were centrifuged at 17,320 *g* at 4°C for 10 min. The supernatants were collected to determine SOD, CAT, and GPx activities using commercial assay kits. The activities of SOD, CAT, and GPx were measured at absorbance of 440, 540, and 340 nm using a CLARIOStar microplate reader (BMG LABTECH, Offenburg, Germany).

### 2.12 Statistical analysis

SPSS19.0 statistical software was used for statistical analysis. All the data were collected in at least three independent experiments and expressed as the mean ± standard deviation (SD). Statistical analysis was carried out using one‐way analysis of variance (ANOVA), followed by Tukey post hoc analysis. The statistical significance was considered at *p* < .05.

## 3 RESULTS

### 3.1 Antioxidant properties, and total phenolic and anthocyanin contents in CNPE

To determine the antioxidant capabilities of CNPE, we also examined the antioxidant activities by DPPH and FRAP assays. The IC50 value of DPPH radical scavenging activity was 7.30 ± 0.07 mg/mL (64.43 ± 2.23 μmol TE/g DW) and the reducing antioxidant power by FRAP assay was 118.71 ± 8.49 μmol TE/g DW (Table 1). The results revealed that CNPE has strong radical scavenging activity while there was a low reducing power activity. Total phenolic and anthocyanin contents were measured by the method described previously (Nantacharoen et al., 2022; Srimard et al., 2022; Sukprasansap et al., 2017). We found that CNPE had high levels of total phenolics (2147.62 ± 1.60 mg GAE/100 g DW) and total anthocyanins (155.71 ± 9.88 mg C3G/100 g DW), as presented in Table 1. Furthermore, we identified the content of the major anthocyanin‐C3G in CNPE to be 96.66 ± 0.31 mg/100 g DW, with 0.32% RSD using HPLC analysis.

**TABLE 1:** Antioxidant activities, total phenolic, total anthocyanin, and C3G contents of CNPE.

DPPH IC50 (mg/mL)	FRAP (μmol TE/g DW)	Total phenolic content (mg GAE/100 g DW)	Total anthocyanin content (mg C3G/100 g DW)	C3G content (mg/100 g DW)
7.30 ± 0.07	118.61 ± 8.49	2147.62 ± 1.60	155.71 ± 9.88	96.66 ± 0.31

*Note*: Data are presented as mean ± SD (*n* = 3).

Abbreviations: C3G, cyanidin‐3‐glucoside; DW, dry weight; GAE, gallic acid equivalent; TE, Trolox.

### 3.2 Effect of CNPE on cell viability in HepG2 cells

**FIGURE 1:**
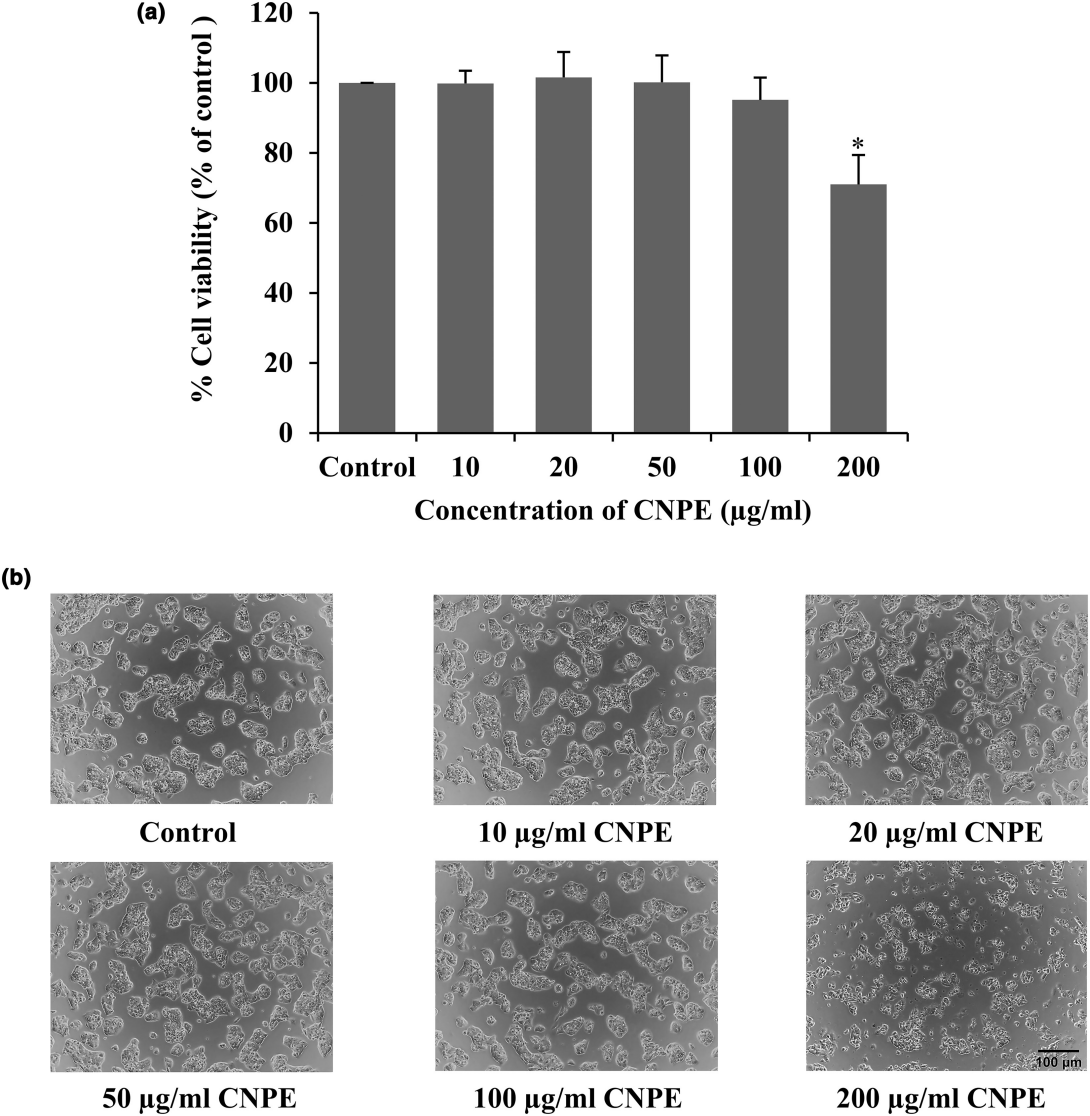
Effect of CNPE on cell viability of HepG2 cells. (a) HepG2 cells were treated with different concentrations (10–200 μg/mL) of CNPE for 24 h, measured using MTT assay. (b) Morphology of HepG2 cells in each of the various concentrations of CNPE, detected by phase‐contrast microscopy at 10× magnification. Scale bar represents 100 μm. Data are represented as mean ± SD, *n* = 3. *p < .05 compared to control group.

CNPE was evaluated for its cytotoxicity in human hepatocellular HepG2 cells using the MTT assay to investigate whether CNPE in various concentrations is non‐cytotoxic or cytotoxic to our cell model. According to the findings of the experiment, CNPE was non‐toxic to cells at concentrations of 10, 20, 50, and 100 μg/mL. Their percentages of cell viability were higher than 80% compared to the control group; there was no significant difference (Figure 1a). Whereas cell viability significantly decreased at a concentration of 200 μg/mL by about 70% compared to the control group, as shown in Figure 1a. Additionally, the morphology of cells was observed by phase‐contrast microscopy in each CNPE concentration. The highest concentration of CNPE, at 200 μg/mL, exhibited shrinkage and reduction of the cell numbers compared to the control and other treatment groups (Figure 1b). Thus, based on this experiment, a maximum concentration of CNPE (100 μg/mL) was used in further experiments.

### 3.3 Effect of H_2_O_2_ on cell viability in HepG2 cells

**FIGURE 2:**
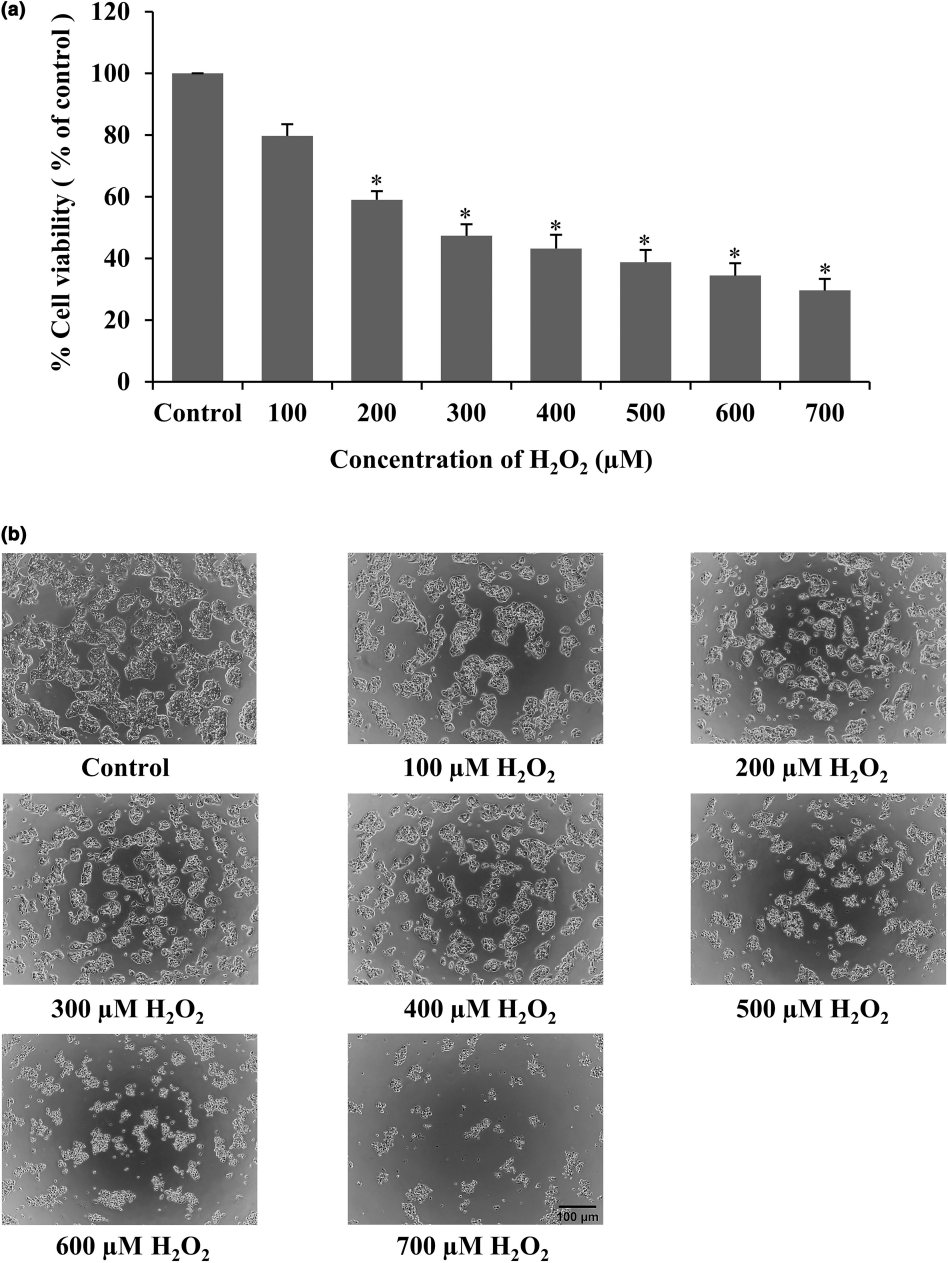
Effect of H_2_O_2_ on cell viability of HepG2 cells. (a) HepG2 cells were treated with various concentrations of H_2_O_2_ (100–700 μM) for 24 h, measured by MTT assay. (b) Morphology of HepG2 cells in each of the various concentrations of H_2_O_2_, observed by phase‐contrast microscopy at 10× magnification. Scale bar represents 100 μm. Data are represented as mean ± SD, *n* = 3. *p < .05 compared to control group.

Next, to investigate the concentration of H_2_O_2_ required to induce cytotoxicity in HepG2 cells, the cells were induced with H_2_O_2_ at various concentrations (100–700 μM) for 24 h using MTT assay. The results of the study showed that a concentration of H_2_O_2_ at 200–700 μM significantly decreased the cell viability compared to control cells (Figure 2a). We found that a concentration of H_2_O_2_ at 300 μM caused an approximately 50% reduction in HepG2 cell viability when compared to the control group (Figure 2a). In addition, the morphology of H_2_O_2_‐treated cells was detected by phase‐contrast microscopy. Figure 2b shows the change in the shape and population of cells. The increasing H_2_O_2_ concentrations affected cell contraction and reduced the number of cells. Therefore, based on this study, a concentration of 300 μM H_2_O_2_ was then selected for use in subsequent investigations of the protective effects of CNPE on H_2_O_2_‐induced HepG2 cells.

### 3.4 CNPE suppresses H_2_O_2_‐induced toxicity in HepG2 cells

**FIGURE 3:**
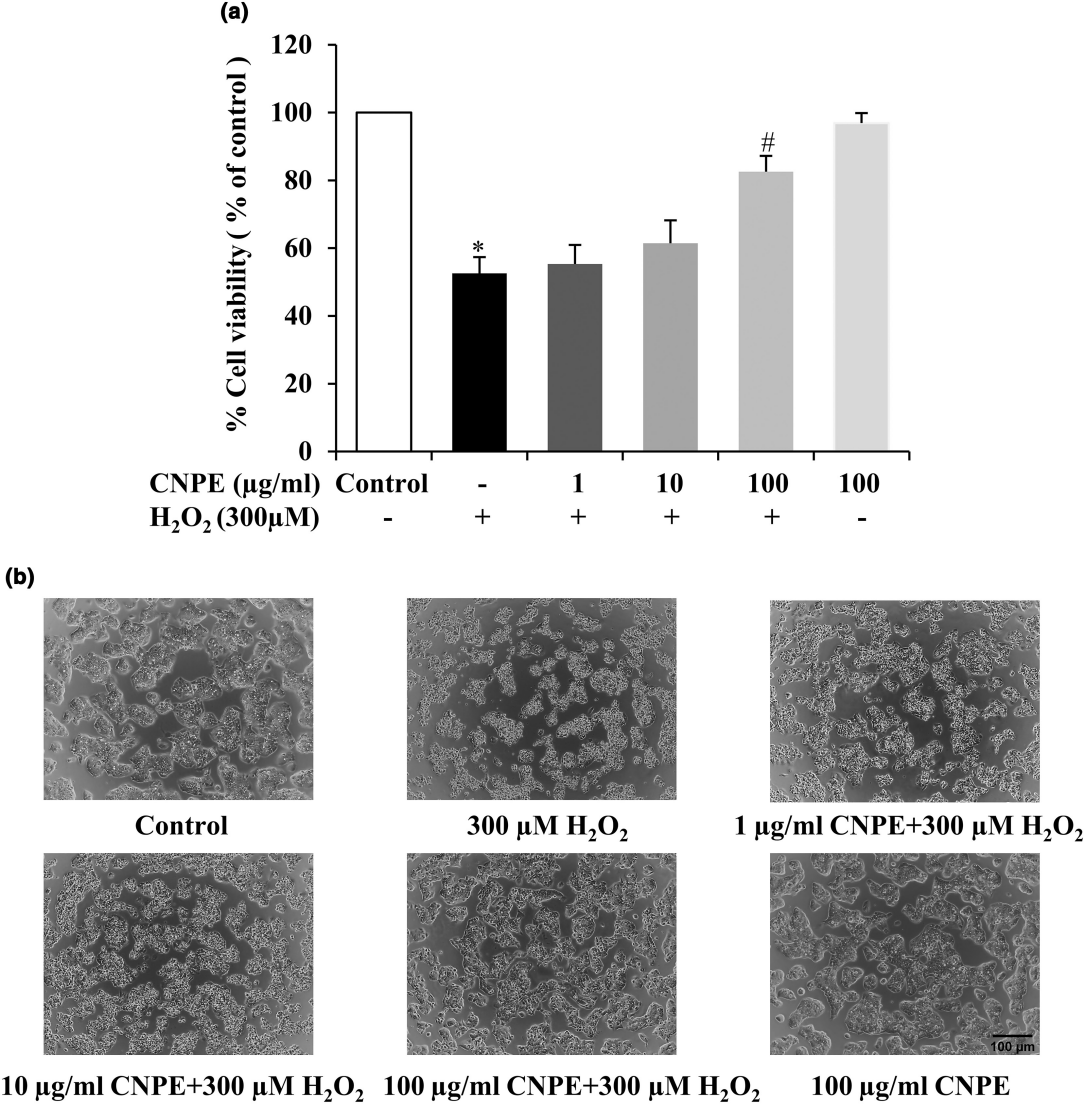
Effect of CNPE on cell viability of H_2_O_2_‐induced toxicity in HepG2 cells. (a) HepG2 cells were pre‐treated with 1–100 μg/mL of CNPE for 24 h, followed by H_2_O_2_ treatment at 300 μM for 24 h. Cell viability was measured using MTT assay. (b) Morphology of cells was examined in each group by phase‐contrast microscopy at 10× magnification. Scale bar represents 100 μm. Data are represented as mean ± SD, *n* = 3. *p < .05 compared to control group, ^#^ *p* < .05 compared to H_2_O_2_‐treated group.

To evaluate the protective effect of CNPE on cell viability in HepG2 cells caused by H_2_O_2_, the cells were pretreated with CNPE at different concentrations (1–100 μg/mL) obtained from the previous experimental results. After incubation for 24 h, cells were incubated with 300 μM H_2_O_2_ for 24 h, then cell viability was assessed by MTT assay. The results showed that pretreating cells with CNPE could protect against H_2_O_2_‐induced toxicity of HepG2 cell viability in a concentration‐dependent manner (Figure 3a). In particular, the pretreatment of cells with CNPE at 100 μg/mL significantly reduced H_2_O_2_‐induced toxicity compared to the H_2_O_2_ treatment group, in which CNPE alone also did not affect the decrease of cell viability. Meanwhile, the cell morphology of pretreated cells with 100 μg/mL CNPE could restore their native morphology and show no marked difference to the control group (Figure 3b). These results demonstrated that the pretreatment of cells with CNPE has an inhibitory effect on H_2_O_2_‐induced cellular toxicity in HepG2 cells.

### 3.5 Evaluation of C3G on cytotoxic and protective effects in HepG2 cells caused by H_2_O_2_

**FIGURE 4:**
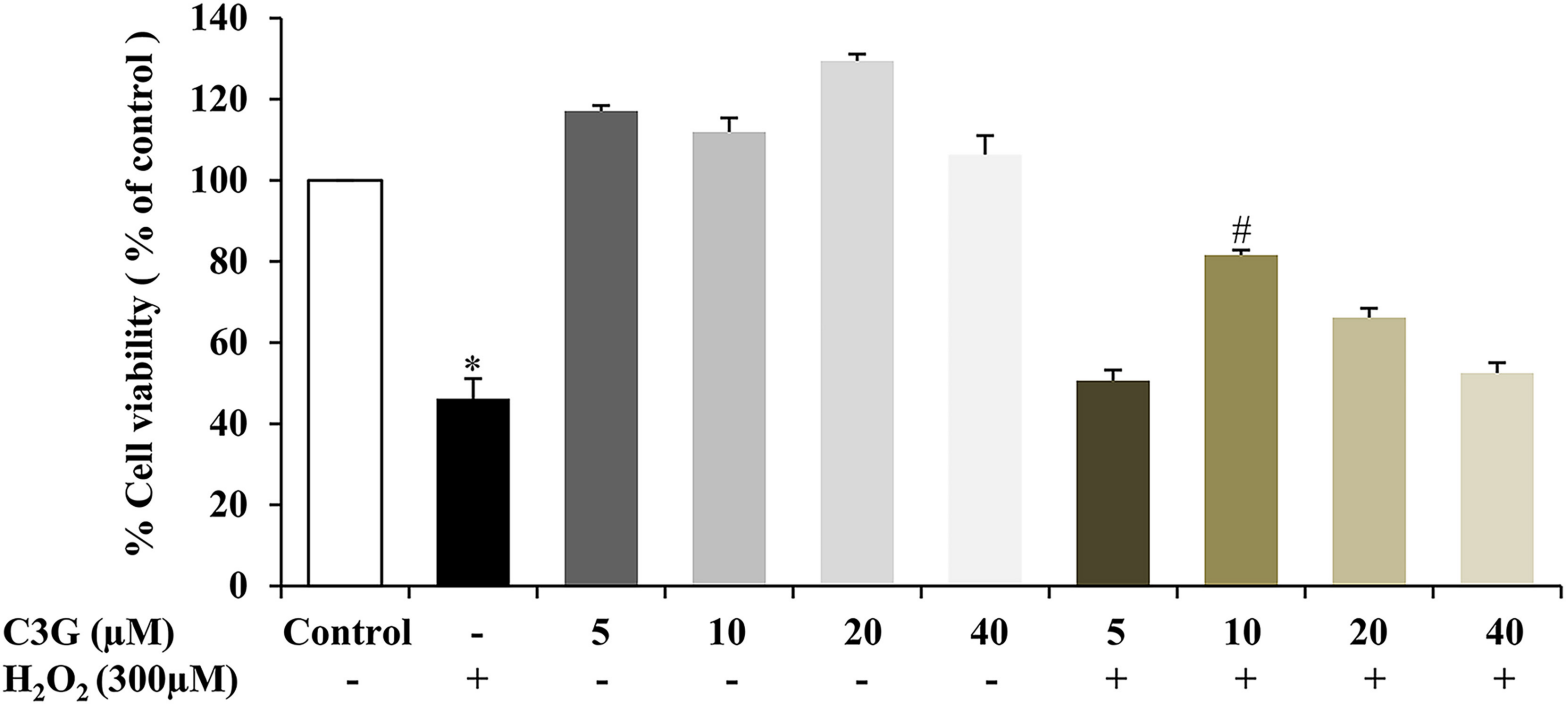
Effect of C3G on cell viability of HepG2 cells. The cells were treated with various concentrations of C3G (5–40 μM) for 24 h, followed by H_2_O_2_ treatment at 300 μM for 24 h. Cell viability was measured using MTT assay. Data are represented as mean ± SD, *n* = 3. *p < .05 compared to control group, ^#^ *p* < .05 compared to H_2_O_2_‐treated group.

C3G was identified and recognized as the major anthocyanin found in the ripe fruit of CNPE in previous studies (Jansom et al., 2008; Sukprasansap et al., 2017). Thus, in this experiment, C3G was also used as a positive control. To determine the cytotoxicity of C3G in HepG2 cells, the cells were treated with C3G (5–40 μM) alone for 24 h or followed by incubating with 300 μM H_2_O_2_ for 24 h. Cell viability was measured by MTT assay. The results showed that cells pre‐treated with C3G at 5–40 μM alone were non‐toxic and did not affect the cell viability of HepG2 cells. However, after incubation with 300 μM H_2_O_2_, the pretreatment of cells with C3G at 5, 20, and 40 μM did not significantly protect HepG2 cells, whereas 10 μM of C3G distinctly prevented the H_2_O_2_‐induced toxicity in the cell model compared to the H_2_O_2_‐treated group (Figure 4). Consequently, the highest protective effect at 10 μM of C3G was chosen for further experiments.

### 3.6 CNPE inhibits H_2_O_2_‐induced caspase activities in HepG2 cells

**FIGURE 5:**
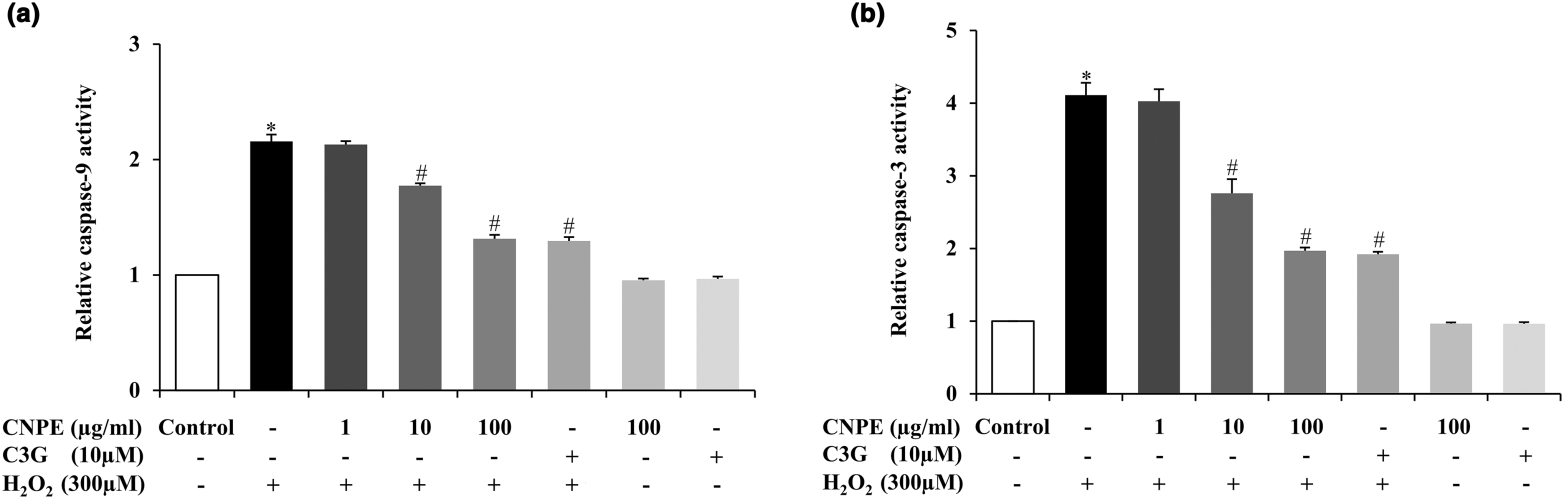
Effect of CNPE on caspase activities in HepG2 cells caused by H_2_O_2_. The cells were pre‐treated with 1–100 μg/mL of CNPE or 10 μM of C3G (used as a positive control) for 24 h, followed by H_2_O_2_ treatment at 300 μM 24 h. Relative (a) caspase‐9 and (b) caspase‐3 activities were assessed by assay kit. Data are represented as mean ± SD, *n* = 3. *p < .05 compared to control group, ^#^ *p* < .05 compared to H_2_O_2_‐treated group.

Cysteine proteases, such as caspase‐9 and caspase‐3, play a central role in the apoptosis process (Yuan et al., 2018). Therefore, to investigate the caspase activity to understand experimental modulation of the apoptotic response on human hepatoma HepG2 cells, the activities of cleaved caspase‐9 and caspase‐3 were detected by assay kit. Cells were pretreated with CNPE at 1–100 μg/mL concentrations or C3G at 10 μM (used as a positive control). After incubation for 24 h, cells were incubated with 300 μM H_2_O_2_ for 24 h. Figure 5 shows H_2_O_2_ alone dramatically increased the levels of cleaved caspase‐9 and caspase‐3 activities compared to the control group. Meanwhile, in the CNPE or C3G alone treatment there was no significant difference compared to the control group (Figure 5a,b). Obviously, pretreating cells with 10–100 μg/mL of CNPE or 10 μM of C3G significantly reduced the level of cleaved caspase‐9 and caspase‐3 activities compared to the H_2_O_2_ treatment group (Figure 5a,b). These results suggest that H_2_O_2_ treatment has the potential to stimulate apoptosis and that CNPE is able to suppress H_2_O_2_‐induced apoptotic death in HepG2 cells.

### 3.7 CNPE represses H_2_O_2_‐induced apoptosis proteins in HepG2 cells

**FIGURE 6:**
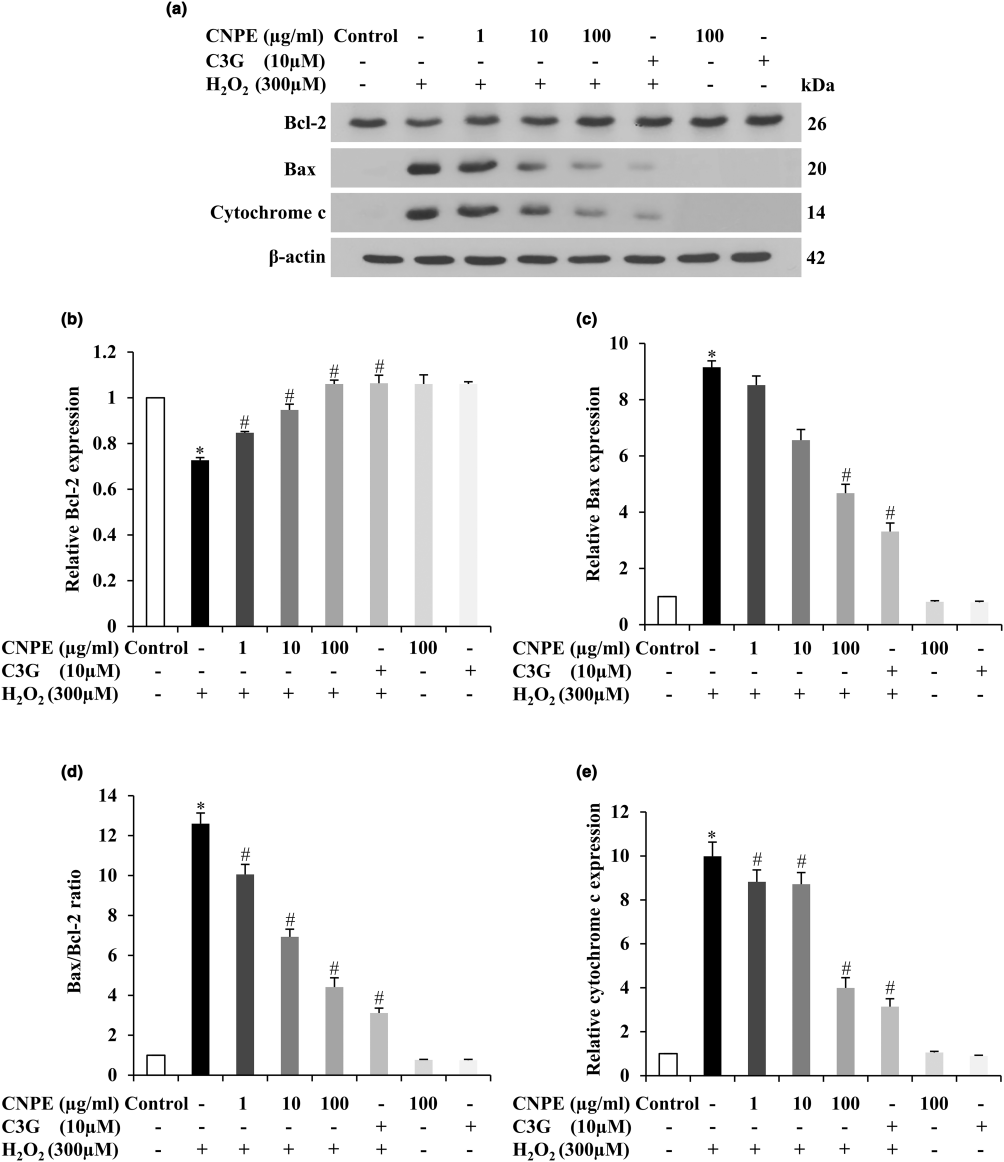
Effect of CNPE on apoptotic proteins in HepG2 cells caused by H_2_O_2_. HepG2 cells were pre‐treated with 1–100 μg/mL of CNPE or 10 μM of C3G (used as a positive control) for 24 h, followed by H_2_O_2_ treatment at 300 μM 24 h. (a) The levels of protein expression were evaluated by Western blot analysis. The histogram graph presents the relative protein expressions of (b) Bcl‐2, (c) Bax, (d) Bax/Bcl‐2 ratio, and (e) cytochrome c, quantified by densitometry and normalized with β‐Actin. Data are represented as mean ± SD, *n* = 3. *p < .05 compared to control group, ^#^ *p* < .05 compared to H_2_O_2_‐treated group.

Apoptosis is a type of specifically programmed cell death. This process is also involved in damaged or dangerous cells and is established at many molecular levels (Green & Kroemer, 2004; Xu et al., 2015). The protein molecules associated with the apoptosis pathway include Bax and cytochrome c (pro‐apoptotic proteins) and Bcl‐2 (anti‐apoptotic protein) (Roduit et al., 2014). The apoptotic protein signal (Bax, Bcl‐2, and cytochrome c) of human hepatoma HepG2 cells was determined by Western blot analysis. The cells were pretreated with CNPE (1–100 μg/mL) or 10 μM C3G (used as a positive control) for 24 h, and then incubated with 300 μM H_2_O_2_ for 24 h. As presented in Figure 6, the H_2_O_2_ treatment alone increased significantly the protein expressions of Bax and cytochrome c but evidently decreased Bcl‐2 expression. Remarkably, the pretreatment of cells with CNPE considerably inhibited the protein expression of Bax and cytochrome c, meanwhile significantly increasing the expression of Bcl‐2 protein in a concentration‐dependent manner compared to the H_2_O_2_ treatment group (Figure 6a–e). In addition, pretreatment with CNPE dramatically decreased the ratio of Bax/Bcl‐2 in a concentration‐dependent manner (Figure 6d). Moreover, C3G alone could significantly suppress the apoptotic protein Bax and cytochrome c and apparently also upregulate the antiapoptotic protein (Bcl‐2) as well as CNPE treatment at the highest concentration (100 μg/mL), as shown in Figure 6. These results indicate that CNPE can protect against H_2_O_2_‐induced oxidative toxicity and cell death via apoptotic protein signals, including Bcl‐2, Bax, and cytochrome c in the apoptosis pathway of HepG2 cells.

### 3.8 CNPE promotes the activities of cellular antioxidant enzymes in HepG2 cells

**FIGURE 7:**
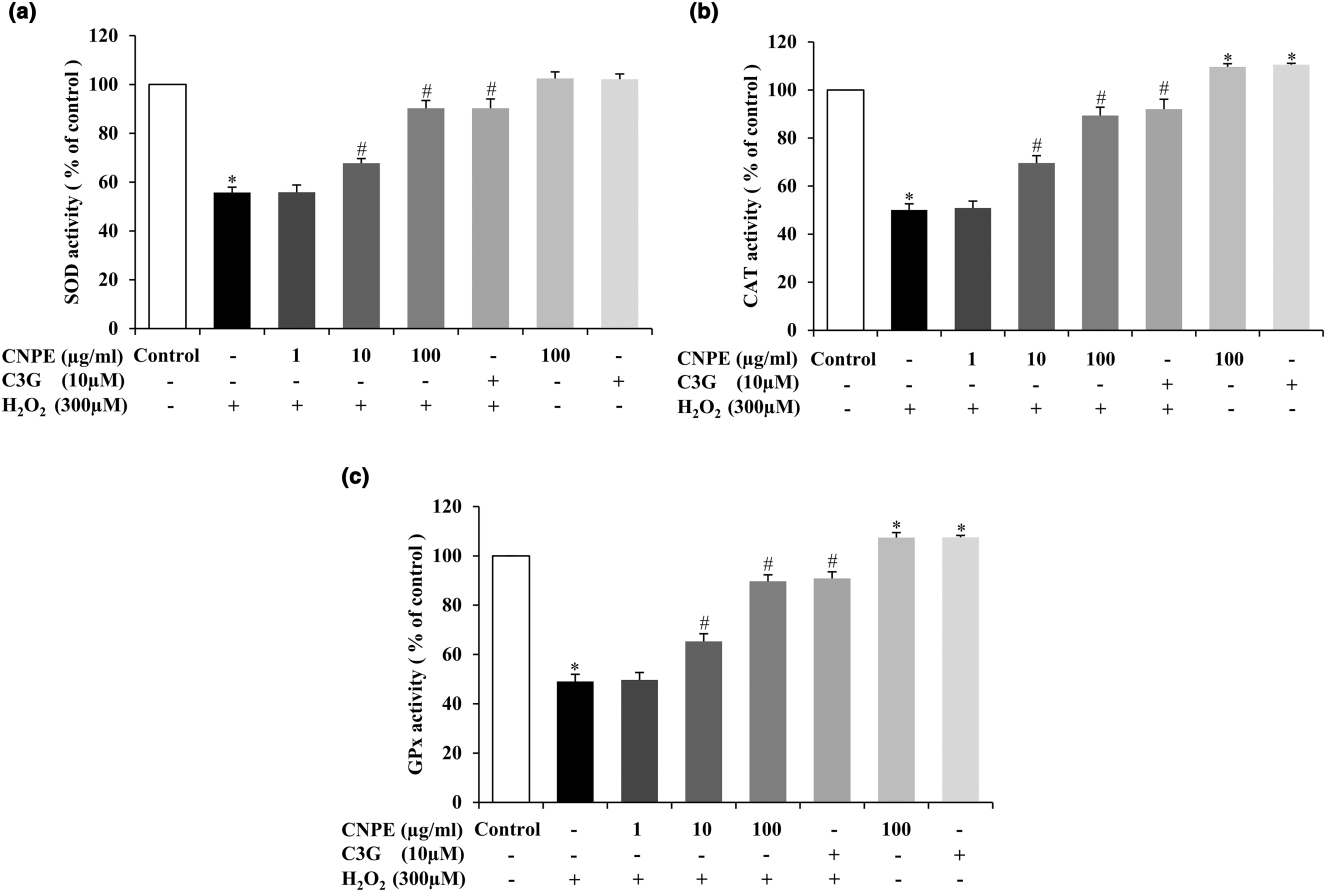
Effect of CNPE on antioxidant enzymes activities in HepG2 cells induced by H_2_O_2_. The cells were pre‐treated with 1–100 μg/mL of CNPE or 10 μM of C3G (used as positive control) for 24 h, followed by H_2_O_2_ treatment at 300 μM 24 h. The activities of antioxidant enzymes, namely (a) SOD, (b) CAT, and (c) GPx were assessed by assay kit. Data are represented as mean ± SD, *n* = 3. *p < .05 compared to control group, ^#^ *p* < .05 compared to H_2_O_2_‐treated group.

The enzymatic antioxidant defense is an important system for preventing and reducing oxidative stress in mammalian cells. Primary enzymatic antioxidants of this defense system, including SOD, CAT, and GPx, can enhance cells' ability to resist oxidative damage caused by H_2_O_2_ (Birangane et al., 2011; Tan et al., 2020). Therefore, to examine the effect of CNPE on the antioxidant enzyme activities in our HepG2 cells, the cells were pretreated with CNPE (1–100 μg/mL) or 10 μM C3G (used as a positive control) for 24 h, followed by 300 μM of H_2_O_2_ for 24 h. As shown in Figures 7a–c, the H_2_O_2_ treatment only significantly decreased the levels of SOD, CAT, and GPx activities compared to the control group. We found that pretreatment of cells with 1–100 μg/mL CNPE or 10 μM C3G tended to increase SOD, CAT, and GPx activities. Interestingly, 100 μg/mL of CNPE significantly showed the highest activity as well as C3G compared to the H_2_O_2_ treatment group of all three enzymes (Figure 7a–c). However, CNPE or C3G alone treatment could increase the levels of CAT and GPx activities (Figure 7b,c), whereas there was no significant difference in the level of SOD activity (Figure 7a) compared to the control group. The results demonstrate that treating cells with CNPE alone could upregulate the antioxidant enzyme activities of CAT and GPx. Moreover, pretreatment of cells with CNPE could enhance the activities of these primary cellular antioxidant enzymes (SOD, CAT, and GPx) compared to H_2_O_2_‐treated group. These findings suggest this extract acts as a potential protective antioxidant against H_2_O_2_‐induced oxidative toxicity and damage in our HepG2 cell model.

## 4 DISCUSSION

Globally, chronic liver diseases in all severity stages of diseases are estimated to be present in 1.5 billion cases. NAFLD is the most common cause of chronic liver disease which is associated with metabolic syndrome such as obesity, diabetes, hyperlipidemia, etc. These accounted for 59% of all causes, followed by HBV (29%), HCV (9%), and ALD, which typically develop in patients with severe disorders of alcohol consumption (2%) and other liver diseases (1%) (Balogh et al., 2016; Embade & Millet, 2017). The majority of chronic liver diseases involve oxidative toxicity, which results in liver damage and loss of liver function (Yuan et al., 2018). The injured cells accumulate over a long time to the point of chronic injury regardless of the risk factors associated with viral hepatitis, ALD, or NAFLD, resulting in oxidative stress in hepatocytes. Although liver cells can regenerate themselves if the cells are injured for a long time, they result in liver macrophages such as kupffer cells and hepatic stellate cells repeating their transformation into myofibroblast‐like cells, contributing to fibrosis in the liver (Ray et al., 2012; Yao et al., 2016). Furthermore, the liver has the capability to produce various radical species, which can lead to increased levels of oxidative stress. If this condition becomes chronic, it stimulates the progression of fibrosis, cirrhosis, and eventually hepatocellular carcinoma (Cheemerla & Balakrishnan, 2021; Michalopoulos & Bhushan, 2021; Sharma & Nagalli, 2021). The overproduction of radicals derived from oxygen such as H_2_O_2_ leads to oxidative toxicity, causing cell damage or even cell death (Kmiec, 2001). H_2_O_2_ is produced by reduction of molecular oxygen to superoxide anion. Then, it is converted into H_2_O_2_, catalyzed by SOD. H_2_O_2_ can give rise to highly toxic hydroxyl radicals through the Fenton reaction (Cuypers et al., 2016).

Currently, natural extracts are found to have beneficial properties in preventing many diseases including chronic liver diseases. Several in vitro and in vivo studies have shown that plant extracts play a role in the inhibition of oxidative stress, especially plant and fruit extracts containing anthocyanin. It was found that the extracts could effectively prevent the harmful effects of oxidative stress in cells (Fallah et al., 2020; Rahman et al., 2008; Wang et al., 2000). Consumption of fruits or berries rich in anthocyanin may prevent the development of chronic liver disease by reducing the reactive radicals and oxidative stress and upregulating the antioxidant defense in cellular system as previously described. In Thailand, the indigenous berry fruit, namely *C. nervosum* var. *paniala*, is found in the Northern region. The local name is Ma‐kiang. The ripe fruit contains abundant anthocyanins. Previous studies found that C3G is the major anthocyanin in the ripe *C. nervosum* var. *paniala* fruits (Jansom et al., 2008; Sukprasansap et al., 2017) which has shown the properties of antioxidant, anti‐inflammation (Tan et al., 2019), anticancer (Cho et al., 2017), and neuroprotection (Sukprasansap et al., 2020). In this study, our results showed that CNPE could scavenge free radicals using DPPH assay and reduce the power activity of ferric iron to ferrous iron by FRAP assay (Table 1). Total phenolic and total anthocyanin contents of CNPE were also discovered (Table 1). These were consistent with the data from previous studies (Nantacharoen et al., 2022; Srimard et al., 2022; Sukprasansap et al., 2017) which showed the strong antioxidant capabilities of CNPE and high total anthocyanin content. Moreover, the major anthocyanin C3G found in this CNPE was determined and identified by HPLC (Table 1). It was higher than the previous report from Sukprasansap et al. (2017). This might correspond to the cultivated areas as well as different locations and annual climate. Furthermore, it is also related to the ripe *C. nervosum* var. *paniala* fruits that we used. They were collected from the cultivated area of the Plant Genetic Conservation Project under the Royal Initiation of Her Royal Highness Princess Maha Chakri Sirindhorn (RSPG), Lampang province, Thailand, where this plant has been improved and developed to be better breeds by RSPG. Moreover, it might involve the extraction solvent and method of extracting the sample with 95% ethanol, as described above in the materials and methods section. This extraction method makes the CNPE more concentrated and dark purple color which results in the C3G content which was higher than previous data from Sukprasansap et al. (2017). They were collected from Maekue, Doi Saket, Chiang Mai province, Thailand, where this plant naturally grows. The ripe fruits were extracted with distilled water and homogenized using a blender according to the method described by Sukprasansap et al. (2017). The aqueous extract had a purplish‐red color. These suggest that the extract obtained from their method has a dilute concentration than our method above. Hence, the different methodology and location result in the C3G content being lower than our presented data. Based on the results, it appears that CNPE exhibits potent and direct antioxidant properties. This indicates that CNPE could potentially play a crucial role in reducing the presence of free radicals. Consequently, it may be able to safeguard cells from oxidative toxicity in the cellular system.

H_2_O_2_ is one of the main constituents of intracellular radical species generated during mitochondrial oxidative metabolism. Excessive H_2_O_2_ leads to the accumulation of oxidative stress, causing cell damage or even cell death (Murphy, 2009; Nindl et al., 2004). Previous studies have used H_2_O_2_‐induced oxidative stress in HepG2 cells (Chiangsom et al., 2019; Xu et al., 2020). In the present study, H_2_O_2_ was also used to induce oxidative stress in our HepG2 cell model. Meanwhile, CNPE was used to study the protective effect against H_2_O_2_‐induced oxidative damage in the cells. Results indicated that CNPE at 10–100 μg/mL was non‐toxic to HepG2 cells (Figure 1), hence the maximum concentration of CNPE (100 μg/mL) was used to further investigate the protective effect against H_2_O_2_‐induced oxidative stress in HepG2 cells. Then, the cells were pretreated with 1, 10, and 100 μg/mL of CNPE for 24 h prior to incubation with 300 μM H_2_O_2_ for 24 h. The results showed that the percentage of cell viability of HepG2 cells significantly decreased when compared to the control group, suggesting that H_2_O_2_ causes oxidative stress and toxicity in HepG2 cells. At the same time pretreated cells with CNPE significantly protected the cell viability of HepG2 cells when compared to the H_2_O_2_ group and CNPE restored the morphology of cells as well as the control group (Figure 3). It has been clearly shown that CNPE can reduce toxicity caused by H_2_O_2_. Several studies, as well as our previous works, indicated that cyanidin‐3‐glucoside or C3G is a major anthocyanin and the key bioactive component found in CNPE, detected by the Electrospray ionization‐mass spectrometric analysis and HPLC technique (Chariyakornkul et al., 2022; Charoensin et al., 2012; Jansom et al., 2008; Sukprasansap et al., 2017). In this report, we have also confirmed and identified this C3G‐bioactive anthocyanin marker by HPLC analysis. Therefore, C3G was used as the positive control and the marker to standardize the extract used to prevent cell damage and death in our study. C3G is the most widespread anthocyanin in nature and the most active antioxidant anthocyanin (Horbowicz et al., 2008; Jansom et al., 2008). Several reports found that C3G protects against oxidative stress‐induced liver damage by decreasing intracellular radicals, and enhancing the antioxidant activities such as SOD, CAT, and GPx (Tan et al., 2020; Yu et al., 2020).

Next, the mechanisms of CNPE on H_2_O_2_‐induced cellular damage in HepG2 cells were investigated. For the apoptosis pathway, this mechanism relates to cell death that includes apoptosis‐related proteins such as Bax, Bcl‐2, cytochrome c, cleaved caspase‐9, and caspase‐3. The apoptotic pathway is activated by both intracellular and extracellular signals via two different pathways, an intrinsic and an extrinsic pathway (Pfeffer & Singh, 2018). In this study, the apoptotic pathway was investigated via an intrinsic pathway in which the apoptosis process occurs in the mitochondria to eliminate cells with damaged DNA and cells with abnormal expression of the oncogene. Mitochondrial outer membrane permeabilization (MOMP) is an essential event in the mitochondrial pathway. MOMP is related to caspase activation, which is an important step in the apoptotic cell death process, controlled by the Bcl‐2 family of proteins including Bax (pro‐apoptotic), Bcl‐2 (anti‐apoptotic), and Cytochrome c proteins (Green & Kroemer, 2004; Lopez & Tait, 2015; Xu et al., 2015). Bcl‐2 family proteins are upregulated in response to apoptotic stress (such as DNA damage) by activated Bax, resulting in mitochondrial membrane permeabilization and release of cytochrome c into the cytosol during apoptosis (Zaman et al., 2014). Cytochrome c binds apoptotic protease‐activating factor 1 (APAF1) and forms a complex with procaspase‐9 called “apoptosome” in which procaspase‐9 is converted into caspase‐9 and activates executioner caspase 3 leading to apoptosis (Tait & Green, 2010; Zaman et al., 2014). The results demonstrated that treatment of HepG2 cells with H_2_O_2_ increased the protein expression levels of Bax and cytochrome c and decreased protein expression levels of Bcl‐2 compared to the control group. Meanwhile, pretreatment of cells with CNPE or C3G clearly inhibited the protein expressions of Bax and cytochrome c, while significantly increasing Bcl‐2 expression compared to the H_2_O_2_ treatment (Figure 6). Our results showed that H_2_O_2_ markedly increased the Bax/Bcl‐2 ratio compared to the control group but the pretreatment with CNPE or C3G prior to incubation with H_2_O_2_ significantly decreased the Bax/Bcl‐2 ratio (Figure 6d). It is possible that the expression of the anti‐apoptotic protein, such as Bcl‐2, suppressed apoptosis by inhibiting the expression of pro‐apoptotic protein, such as Bax, then blocking the release of cytochrome c (Hotchkiss et al., 2009; Wang & Lin, 2013). In addition, we further investigated the activation of caspases, namely cleaved caspase‐9 and caspase‐3 which play a central role in the apoptosis process. The results showed that H_2_O_2_ alone significantly increased the cleaved caspase‐9 and caspase‐3 activities compared to the control group. Meanwhile, pretreating cells with CNPE or C3G dramatically suppressed caspase‐9 and ‐3 activities compared to the H_2_O_2_ group. These results suggest that CNPE could inhibit the activation of cleaved caspase‐9 and caspase‐3 (Figure 5a,b). These results indicate that CNPE could reduce cell death by inhibiting the activation of the intrinsic apoptosis pathway. Several studies revealed that H_2_O_2_ can induce oxidative stress and apoptotic cell death, involving an increase in reactive species production, decreasing antioxidant protein activity. However, pretreatment of cells with a purple rice extract containing cyanidin decreased the expression of Bax, cytochrome c, cleavage caspase‐9, and cleavage caspase‐3 and increased the ratio of Bcl‐2/Bax in a concentration‐dependent manner (Thummayot et al., 2014). Importantly, C3G could upregulate the relative protein expression levels of anti‐apoptotic such as Bcl‐2 in HepG2 cells (Tan et al., 2020).

Normally, the underlying mechanism of H_2_O_2_‐induced oxidative and cellular toxicity is related to a decrease in the activities of primary antioxidant enzymes (SOD, CAT, and GPx). The present study showed that the levels of three antioxidant enzyme activities significantly reduced in the H_2_O_2_‐treated cells alone compared to the control group. On the other hand, pretreatment of cells with CNPE or C3G demonstrated a significant increase in the level of antioxidant activities (SOD, CAT, and GPx) compared to the H_2_O_2_ group. Moreover, pretreating cells with CNPE or C3G alone, there was no significant difference in SOD activity, but there was a significant increase in CAT and GPx activities compared to control group. Based on these results, it is possible that CNPE directly increases levels of CAT and GPx activities (Figure 7). This is consistent with previous experiments where it was found that CNPE containing an anthocyanin such as C3G could enhance gene expression of cellular antioxidant enzymes including SOD, CAT, and GPx in neuronal cells (Nantacharoen et al., 2022; Sukprasansap et al., 2020). Furthermore, it was found that C3G could increase the Nrf2 mRNA expression and enhance SOD, CAT, and GPx activities in renal cells of mice with diabetic nephropathy (Qi et al., 2020).

In addition, an in vivo study demonstrated that *C. nervosum* var. *paniala* could help to protect liver of rats from oxidative stress and enhance the antioxidant activities such as GPx, CAT, and heme oxygenase in the liver of rats caused by carcinogens (Taya, 2010). Some evidence reported the inhibitory effect of *C. nervosum* var. *paniala* pulp on acetaminophen (APAP)‐induced acute hepatotoxicity in rats. It found that *C. nervosum* var. *paniala* had high amount of anthocyanin content and free radical scavenging activity, which could suppress oxidative stress from APAP‐induced acute hepatotoxicity and stimulate the antioxidant activities similar to GPx in rat livers (Chariyakornkul et al., 2022). The main anthocyanin of the ripe *C. nervosum* var. *paniala* fruits is C3G as previously described. Some data from in vitro and in vivo studies revealed that C3G could reduce oxidative stress and apoptosis caused by H_2_O_2_‐induced oxidative damage in HepG2 cells and CCl_4_‐induced acute liver damage in mice liver tissue. These relate to the repression of the apoptosis pathway by decreasing the expression of Bax and cleavage caspase‐3 while increasing the expression of Bcl‐2 and the antioxidant enzyme activities such as SOD and GPx (Yu et al., 2020). Furthermore, C3G suppressed hepatic oxidative stress that leads to the activation of inflammation and steatosis, including improving systemic glucose metabolism or preventing hepatic lipid accumulation via regulating the function and the secretion of adipokines from brown adipose tissue in mice receiving a high‐fat diet which led to NAFLD (Li et al., 2020; Pei et al., 2018). The above results are consistent with our study. This may further support our study, that CNPE may help prevent chronic liver disease, whether it is a risk factor for NAFLD or not. Therefore, our findings in this present study suggest that CNPE could protect against oxidative stress by increasing endogenous antioxidant enzyme activities. It is possible that the C3G‐anthocyanin found in CNPE could help to protect cells from oxidative stress caused by H_2_O_2_ and improve antioxidant activities such as SOD, CAT, and GPx in HepG2 cells. Additionally, the synergistic effects of a crude extract of CNPE might promote, at least in part, the antioxidant mechanism and inhibit the apoptosis pathway in HepG2 cells. Overall, this investigation indicated that ripe fruit extract from *C. nervosum* var. *paniala* can prevent liver cell damage and death from oxidative toxicity via the underlying mechanisms of reducing the apoptosis pathway and enhancing the cellular antioxidant enzyme activities in HepG2 cells.

## 5 CONCLUSION

**FIGURE 8:**
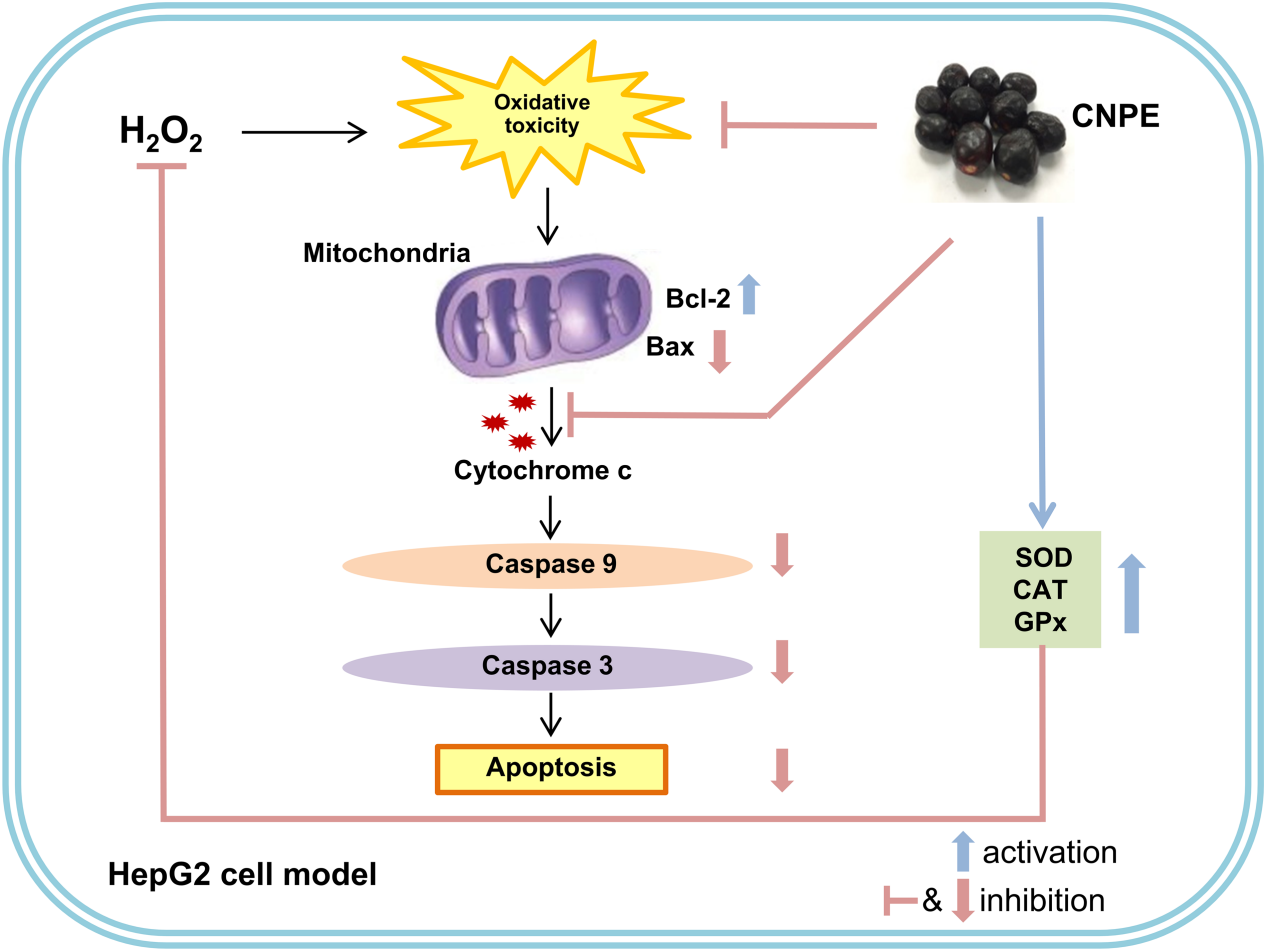
The schematic of the proposed mechanism of CNPE. The protective effects and mechanisms of CNPE against H_2_O_2_‐induced oxidative toxicity and cell death through inhibition of apoptosis pathway and activation of cellular antioxidant enzyme activities in HepG2 cells. Bax, Bcl‐2‐associated X protein; Bcl‐2, B‐cell lymphoma‐2; CAT, catalase; CNPE, *Cleistocalyx nervosum* var. *paniala* fruit extract; GPx, glutathione peroxidase; H_2_O_2_, hydrogen peroxide; SOD, superoxide dismutase.

In summary, CNPE exhibited strong radical scavenging activity and had high amounts of total phenolic and anthocyanin contents. The results demonstrate that CNPE decreases H_2_O_2_‐induced oxidative toxicity in HepG2 cells. Interestingly, CNPE inhibits H_2_O_2_‐induced oxidative damage and apoptosis in HepG2 cells by increasing the viability of cells, the expression of antiapoptotic Bcl‐2 protein, and the activities of cellular antioxidant enzymes, namely SOD, CAT, and GPx. Furthermore, CNPE also suppressed the expressions of apoptotic proteins (Bax and cytochrome c) and the activities of cleaved caspase‐9 and caspase‐3 caused by H_2_O_2_ in our cell model. Likewise, the results from C3G treatment (positive control group) showed a protective effect and mechanism against H_2_O_2_‐induced oxidative damage and apoptosis in HepG2 cells that was similar to the highest concentration of CNPE. Collectively, these results indicate the protective effects of CNPE against H_2_O_2_‐induced oxidative damage and cell death through inhibition of the apoptosis pathway and activation of cellular antioxidant enzyme activities in HepG2 cells. Figure 8 presents a schematic of the proposed mechanism of CNPE in our cell model, HepG2 cells.

## AUTHOR CONTRIBUTIONS

**Pasitta Panritdum:** Conceptualization (equal); data curation (lead); investigation (lead); methodology (lead); writing – original draft (lead). **Chawanphat Muangnoi:** Conceptualization (equal); supervision (equal); validation (equal). **siriporn Tuntipopipat:** Supervision (equal). **Somsri Charoenkiatkul:** Supervision (equal). **Monruedee Sukprasansap:** Conceptualization (equal); data curation (equal); funding acquisition (lead); validation (equal); writing – original draft (equal); writing – review and editing (lead); supervision (equal).

## FUNDING INFORMATION

This research was supported by the National Research Council of Thailand (Grant No. 2018NRCT32900/695259).

## CONFLICT OF INTEREST STATEMENT

The authors declare no conflict of interest.

## Data Availability

Data will be made available on request.
